# Thousands of microbial genomes shed light on interconnected biogeochemical processes in an aquifer system

**DOI:** 10.1038/ncomms13219

**Published:** 2016-10-24

**Authors:** Karthik Anantharaman, Christopher T. Brown, Laura A. Hug, Itai Sharon, Cindy J. Castelle, Alexander J. Probst, Brian C. Thomas, Andrea Singh, Michael J. Wilkins, Ulas Karaoz, Eoin L. Brodie, Kenneth H. Williams, Susan S. Hubbard, Jillian F. Banfield

**Affiliations:** 1 Department of Earth and Planetary Science, University of California, Berkeley, California 94720, USA; 2 Department of Plant and Microbial Biology, University of California, Berkeley, California 94720, USA; 3 School of Earth Sciences and Department of Microbiology, The Ohio State University, Columbus, Ohio 43210, USA; 4 Earth and Environmental Sciences, Lawrence Berkeley National Laboratory, Berkeley, California 94720, USA

## Abstract

The subterranean world hosts up to one-fifth of all biomass, including microbial communities that drive transformations central to Earth's biogeochemical cycles. However, little is known about how complex microbial communities in such environments are structured, and how inter-organism interactions shape ecosystem function. Here we apply terabase-scale cultivation-independent metagenomics to aquifer sediments and groundwater, and reconstruct 2,540 draft-quality, near-complete and complete strain-resolved genomes that represent the majority of known bacterial phyla as well as 47 newly discovered phylum-level lineages. Metabolic analyses spanning this vast phylogenetic diversity and representing up to 36% of organisms detected in the system are used to document the distribution of pathways in coexisting organisms. Consistent with prior findings indicating metabolic handoffs in simple consortia, we find that few organisms within the community can conduct multiple sequential redox transformations. As environmental conditions change, different assemblages of organisms are selected for, altering linkages among the major biogeochemical cycles.

## Abstract (web summary)

Microorganisms from the terrestrial subsurface are understudied. Here, Anantharaman *et al*. analyse aquifer sediments and groundwater by genome-resolved metagenomics and reconstruct 2,540 genomes representing the majority of known bacterial phyla as well as 47 new phylum-level lineages.

The terrestrial subsurface is the largest reservoir of carbon on earth, containing 14–135 Pg of carbon^1^ and 2–19% of all biomass^2^. Microorganisms drive organic and inorganic compound transformations in this environment and thereby control biogeochemical cycles. Our current knowledge of the microbial ecology of the subsurface is primarily based on 16S ribosomal RNA (rRNA) gene sequences. Recent estimates show that <8% of 16S rRNA sequences in public databases derive from subsurface organisms^3^ and only a small fraction of those are represented by genomes or isolates. Thus, there is remarkably little reliable information about microbial metabolism in the subsurface. Further, little is known about how organisms in subsurface ecosystems are metabolically interconnected. Some cultivation-based studies of syntrophic consortia^4^,^5^,^6^ and small-scale metagenomic analyses of natural communities^7^,^8^,^9^ suggest that organisms are linked via metabolic handoffs: the transfer of redox reaction products of one organism to another. However, no complex environments have been dissected completely enough to resolve the metabolic interaction networks that underpin them. This restricts the ability of biogeochemical models to capture key aspects of the carbon and other nutrient cycles^10^. New approaches such as genome-resolved metagenomics, an approach that can yield a comprehensive set of draft and even complete genomes for organisms without the requirement for laboratory isolation^7^,^11^,^12^, have the potential to provide this critical level of understanding of biogeochemical processes.

In this study, we use terabase-scale shotgun DNA sequencing to extensively sample microbial genomes from an aquifer adjacent to the Colorado River, located near Rifle, CO, USA. Previous studies of this aquifer characterized specific lineages of microorganisms, primarily as part of an investigation into the potential for addition of uranium into the subsurface to stimulate uranium immobilization^13^,^14^,^15^,^16^,^17^,^18^,^19^. Here our goal is the extensive recovery of near-complete and complete genomes to enable accurate reconstruction of metabolism and ecological roles of the microbial majority, including previously unstudied lineages. To maximize recovery of genomes, we study 15 geochemically distinct sediment and groundwater environments, some of which were altered via *in situ* manipulation experiments. Our results show that terabase-scale metagenomics can be used as a high-throughput tool to recover thousands of high-quality strain-resolved genomes from a complex subsurface ecosystem. We use these genomes to track dynamics in community composition and metabolic potential across the studied spectrum of environment types, and detect organisms from the ‘rare biosphere'^20^, which may represent as little as <0.001% of a community. Given identification of many new putative phylum-level groups, our metabolic analyses span an unprecedented level of phylogenetic diversity. Our genome-resolved studies at the community-level support the idea that inter-organism interactions are key to turning the globally relevant subsurface biogeochemical cycles of carbon, nitrogen, sulfur and hydrogen.

## Results

### Sampling microorganisms from the terrestrial subsurface

**Figure 1:**
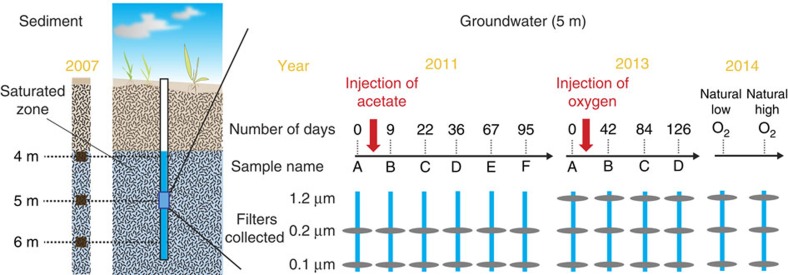
Sampling scheme for sediment and groundwater microbial communities from the Rifle Integrated Field Research site. Samples were collected for metagenomics from sediment and groundwater spanning several redox transitions including natural unamended samples, and acetate and oxygen stimulation of groundwater microbial communities. Sediment samples were collected from depths of 4, 5 and 6 m below the surface. Groundwater was pumped from a depth of 5 m and filtered through serial 1.2, 0.2 and 0.1 μm filters. Groundwater samples were collected at six time points (A–F) during acetate stimulation, four time points during oxygen stimulation (A–D) and two time points representing naturally encountered high (high O_2_)- and low (low O_2_)-oxygen concentrations in the aquifer respectively. 1.2 μm filters from the acetate stimulation experiment were not sequenced.

We used genome-resolved metagenomics to study sediment and groundwater-associated bacteria and archaea from a shallow sediment-hosted perennially suboxic/anoxic aquifer adjacent to the Colorado River, near Rifle, CO, USA^7^,^13^,^14^,^16^,^17^,^21^,^22^. Sediments were collected from a core from depths of 4, 5 and 6 m below ground surface in the saturated zone (Fig. 1; Supplementary Data 1). In addition, groundwater from a depth of 5 m was sequentially filtered onto 1.2, 0.2 and 0.1 μm filters. Four sample sets were collected during an 18-week long experiment in which oxygen-saturated water was injected into the aquifer^23^ and six sample sets derived from an acetate injection experiment conducted over a period of 14 weeks^17^. We also sampled groundwater during naturally encountered low and high oxygen conditions (Fig. 1; Supplementary Data 1).

In total, we sequenced 33 samples and generated 4.58 billion paired-end Illumina sequencing reads, which were assembled into ∼30 Gbp of scaffolds (Supplementary Data 2). Reconstruction of individual genomes was performed by binning on the basis of GC content, tetranucleotide signatures^24^, variance of abundance patterns across individual samples^25^ and taxonomic affiliation of encoded genes in ggKbase (http://ggkbase.berkeley.edu). All genomes were curated to remove wrongly assigned scaffolds, eliminate scaffolding errors and increase scaffold lengths. To enable comprehensive and accurate characterization of microbial metabolic potential, we targeted microorganisms with an initial genome-completion estimate >70% for further analysis (Supplementary Data 3). Ultimately, we generated and analysed 2,516 bacterial genomes (Supplementary Data 4) and 24 archaeal genomes (Supplementary Data 5). Twenty-one of these bacterial genomes are complete (closed, no gaps). Since analysis of strain variations in these genomes was not a goal of this specific study, we clustered the genomes at an average nucleotide identity of 98% (Methods). Using these thresholds, the 2,540 genomes were assigned to 1,297 clusters representing distinct microorganisms (Supplementary Data 6). The genomes have a median genome-completion estimate of >93%. In total, these 1,297 genomes account for up to 29% of all microorganisms detected in groundwater samples and 36% of those from sediments at the site to date, including prior studies (Supplementary Fig. 1). To the best of our knowledge, this is the most detailed genomic sampling of any terrestrial ecosystem. The vast majority of these reconstructed genomes belonged to previously unknown and little studied bacterial and archaeal lineages.

### Phylogenetic diversity and 47 new phylum-level lineages

**Figure 2:**
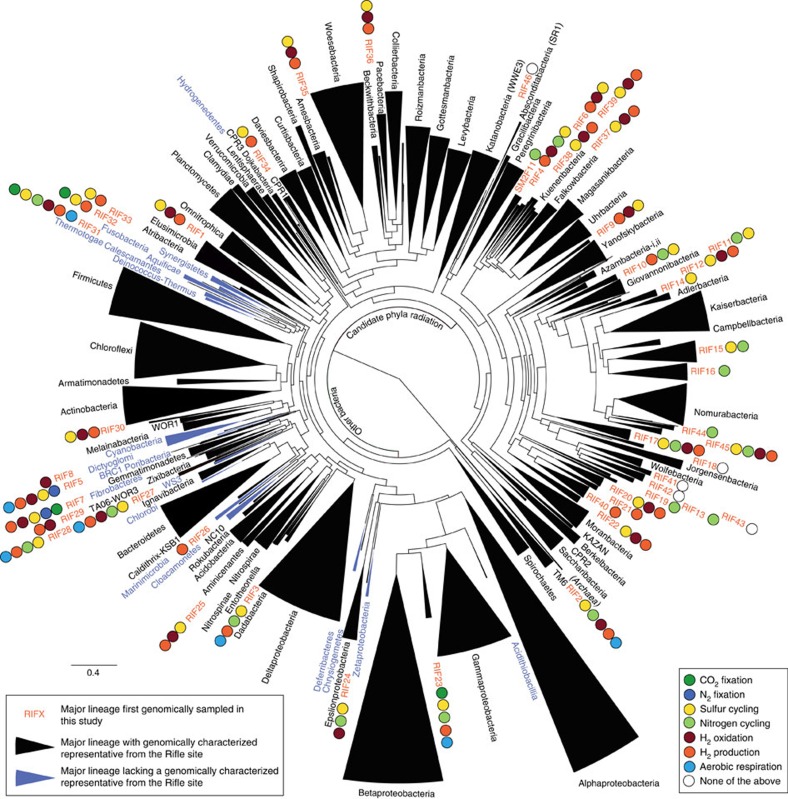
Phylogeny of bacterial genomes inferred by maximum likelihood. The phylogenetic tree is based on 16 concatenated RPs and was collapsed at the phylum level. Colours of the wedges indicate the following: black: phylum-level lineage identified at Rifle; blue: phylum-level lineage not identified at Rifle. Coloured circles describe important biogeochemical roles inferred for newly described phylum-level lineages. Proposed names for newly described phylum-level lineages (RIF1-RIF46 and SM2F11) are detailed in Table 1. The phylogenetic inference configurations with detailed branch support values are provided in Supplementary Fig. 2 and Supplementary Data 12.

To evaluate the phylogeny of the recovered organisms, we performed analyses utilizing both concatenated ribosomal proteins (RPs) and 16S rRNA genes. For the RP tree, we used a previously benchmarked set of 16 RPs that are encoded by genomically co-located genes^26^. Novelty of phylum-level lineages relied upon these phylogenies and previously suggested evolutionary distance metrics^27^ (Methods). The bacterial genomes derive from ∼78% of previously established phylum-level lineages (including candidate phyla; Fig. 2; Supplementary Fig. 2) and from 47 new putative phylum-level lineages (defined using 554 genomes), 46 of which had not been previously detected by 16S rRNA sequencing. Thirty of these new phylum-level lineages belong to the recently described Candidate Phyla Radiation (CPR)^17^ and two affiliate with the *Proteobacteria* (Fig. 2; Table 1). In total, these novel lineages (if validated as phyla by further research) would expand the number of lineages in the Bacterial domain by ∼50% (ref. 28). Less than 11% of all genomes belonged to the four phylum-level lineages that constitute the vast majority of genomes currently in public databases^29^, namely, *Proteobacteria*, *Actinobacteria*, *Firmicutes* and *Bacteroidetes*. Overall, the genomes reported here belong to 117 distinct bacterial and archaeal phylum-level lineages (Supplementary Data 7).

### Estimation of microbial abundance

**Figure 3:**
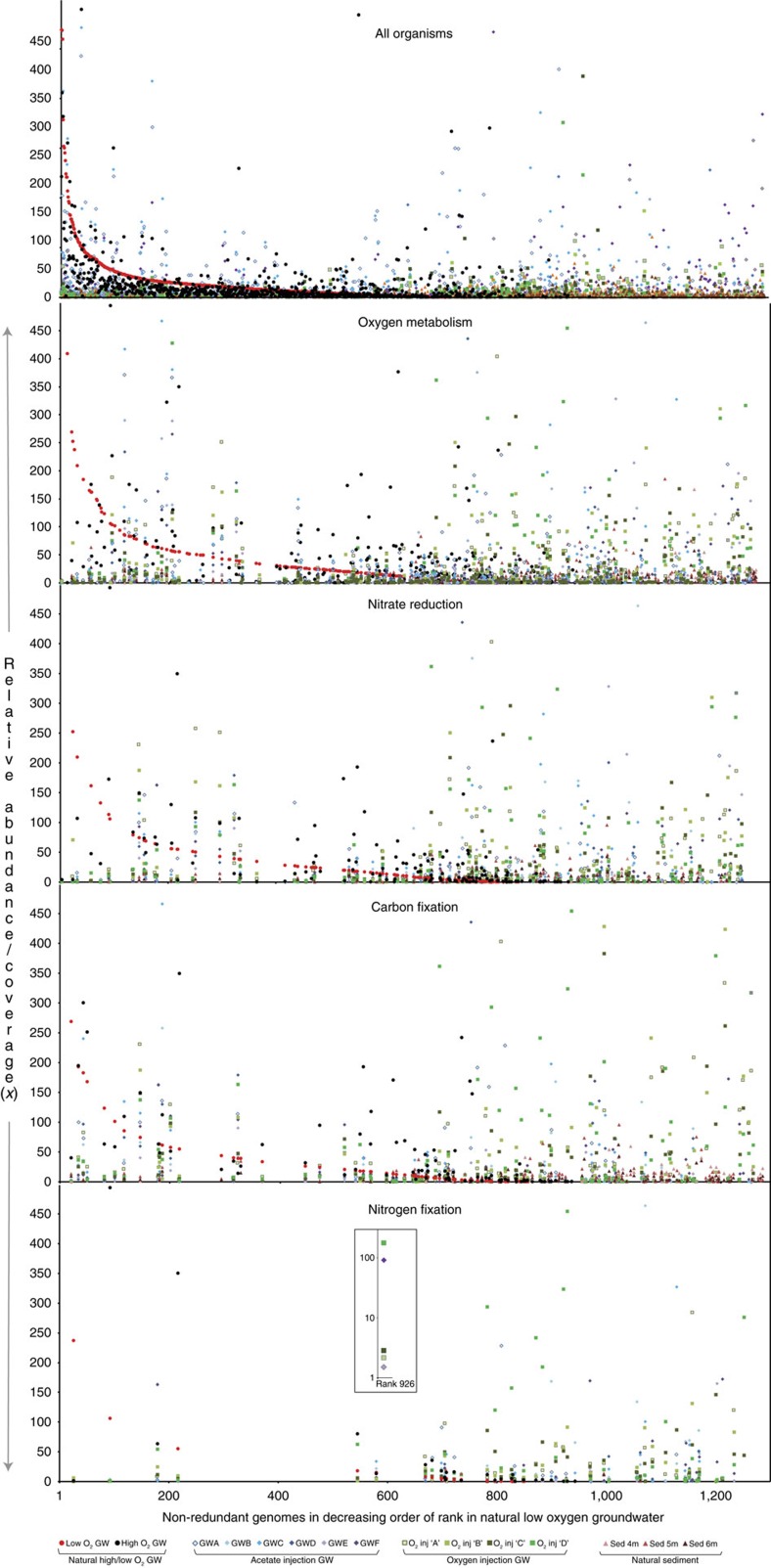
Rank abundance plots highlighting organisms putatively involved in geochemical cycling across 15 different geochemical regimes in the aquifer. Rank abundance curves were computed using whole-genome coverage estimated by read mapping. Organisms with genome coverage greater than 500x are not shown. Symbols represent different perturbations/sample sources: circles: natural high/low-oxygen groundwater; diamonds: acetate injection into groundwater; squares: oxygen injection into groundwater; triangles: natural unamended sediment. *Y* axis represents the normalized relative abundance in the community (genome coverage normalized to the natural low-oxygen groundwater sample). Panels representing specific metabolisms (oxygen metabolism, nitrate reduction, carbon fixation and nitrogen fixation) only show organisms inferred to have that capacity. Inset figure highlights the variation in abundance of a single *Sulfuricurvum* species (*Sulfuricurvum* sp. RIFOXYD12_FULL_44_77) that appears to be able to fix carbon and nitrogen, across the different geochemical conditions. GW, groundwater. For groundwater, only samples collected on the 0.2 μm filters are shown.

We tracked the abundances of each microorganism in communities across 15 distinct geochemical regimes (Supplementary Data 8). Sediments (which include pore fluids) show very high levels of organism diversity yet exhibit more consistency in terms of overall community composition than the extracted pore fluids (natural groundwater; Supplementary Movies 1–5). Changes in environmental conditions appear to drive selection of pore fluid-associated species from the particle-associated ‘microbial seed bank'^30^,^31^. Notably, although organisms capable of specific key processes such as aerobic respiration, nitrate reduction, carbon fixation and nitrogen fixation are present in all samples, the abundant species with these capacities in each environment are typically always different (Fig. 3; Supplementary Data 3). Key ecosystem functions occur in a vast array of genomic contexts (Supplementary Data 9).

### Genome-specific metabolic reconstructions

**Figure 4:**
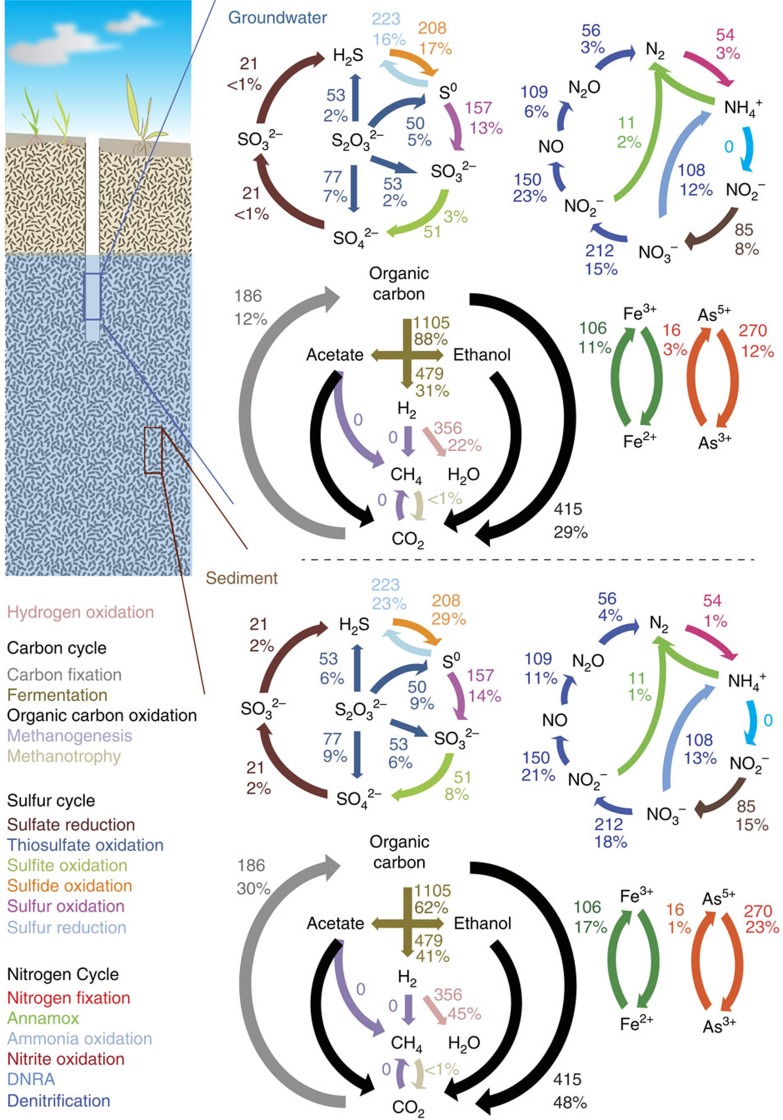
Biogeochemical cycling capacity inferred for the microbial communities in sediment and groundwater in the aquifer. The cycles of C, N, S, H, Fe and As are described above. Colours represent different parts of the individual cycles. Arrows indicate specific transformations. Numbers and percentages on arrows indicate the number of organisms inferred to be able to perform the transformation, and their total relative abundance in the microbial community, respectively. For groundwater, only natural unamended samples collected on the 0.2 μm filter were considered. DNRA, dissimilatory nitrate reduction to ammonium.

The 2,540 reconstructed genomes encoded a total of 4,107,178 protein-coding genes. Detailed genome-specific metabolic potential was determined by profiling all the genes against specific databases (KEGG, Uniref, TIGRfam, Pfam and Custom)^32^,^33^,^34^,^35^ using hidden Markov models (HMM)^36^ and homology-based searches^37^ (Methods). Specifically, we targeted genes involved in microbial energy metabolism (electron donors and acceptors), key ecosystem functions such as carbon and nitrogen fixation and other important functions (Supplementary Data 9). Our results show that the use of an inorganic compound as an energy source (lithotrophy) appears to be a common metabolic strategy in the studied subsurface ecosystem (Fig. 4). Across all environments sampled, between 26 and 36% of the genomes analysed carried the potential to use carbon monoxide (CO), hydrogen (H_2_) or reduced sulfur species as electron donors (Supplementary Data 9). Thus, the metabolism of subsurface-associated microbes appears to be closely linked to the biogeochemical cycles of carbon, hydrogen and sulfur. The potential for nitrite and iron transformations is encoded in many fewer genomes, and for methane and ammonia oxidation only rarely (Supplementary Data 9). However, the capacity for anaerobic ammonium oxidation (Anammox), a process rarely observed in subsurface environments^38^, was encoded in a few genomes of members of the phylum *Planctomycetes*. Importantly, the possibility that CO and H_2_ are significant ‘currencies' in the subsurface microbial economy is not evident from the geochemical data, as the concentrations of these compounds are extremely low (<1 mg l^−1^ CO and 2–17 nM H_2_). In combination, the results suggest rapid cycling of CO and H_2_, possibly in syntrophic microbial associations.

Less than 2% of the genomes are predicted to encode the capacity for use of sulfate as an electron acceptor. Oxygen and nitrate appear to be the most widely used terminal electron acceptors, with genes for these functions in 34% and 17% of the genomes, respectively. Nitrite also appears to be a relatively important electron acceptor, and some organisms can potentially convert it to nitric oxide and others to ammonia. Selection for aerobes and denitrifiers is probably a consequence of electron donor availability and proximity to the water table. Overall, the availability of statistics describing prevalence of traits associated with carbon, nitrogen, sulfur and hydrogen cycling (Supplementary Data 9,10) will serve as a benchmark for comparative studies involving other ecosystems.

### Metabolic handoffs in subsurface microbial communities

**Figure 5:**
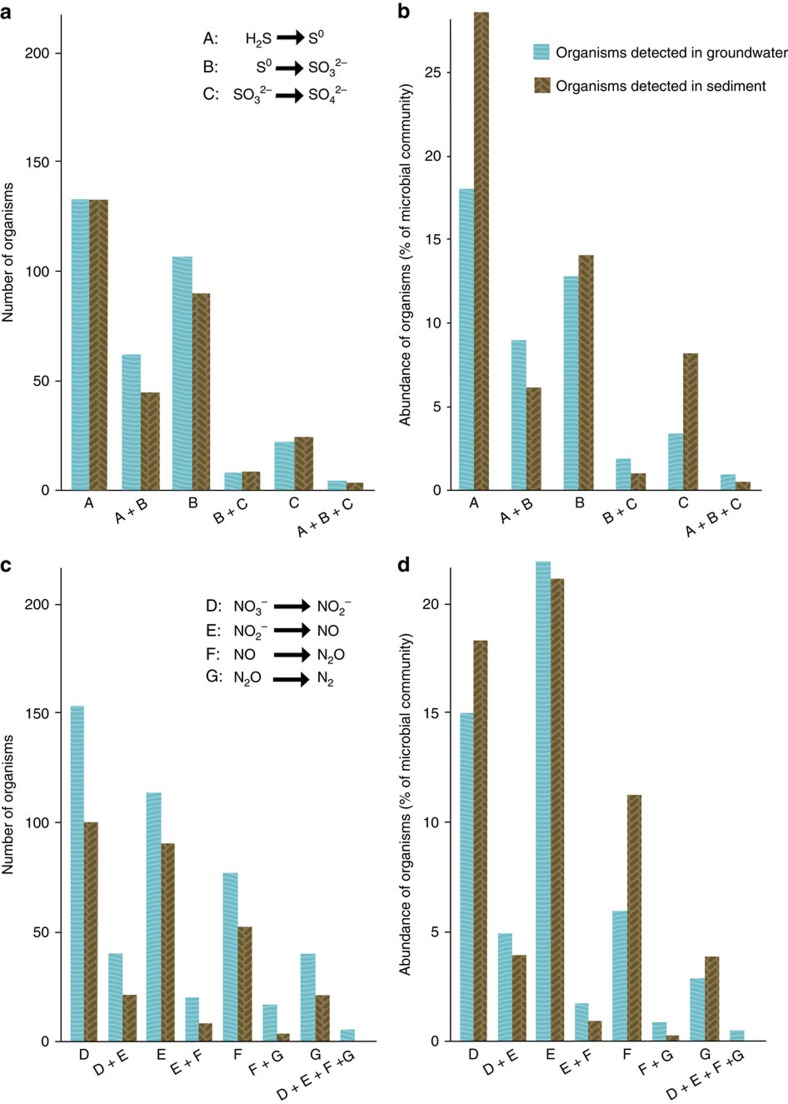
Number and abundance of organisms putatively involved in sequential redox transformations. (**a**,**b**) Number (**a**) and relative abundance (**b**) of organisms inferred to be involved in sequential oxidation of sulfide to sulfate. (**c**,**d**) Number (**c**) and relative abundance (**d**) of organisms putatively involved in sequential reduction of nitrate to N_2_ (denitrification). Only organisms detected at >0.01% of the microbial community were considered. For groundwater, only natural unamended samples collected on the 0.2 μm filter were considered. Organisms considered for step ‘E' (NO_2_^−^→NO) might detoxify NO_2_^−^.

We analysed which metabolic traits are potentially encoded in each genome (Supplementary Data 9). We found that few organisms appear to have the potential for complete oxidation of sulfide to sulfate, or complete denitrification of nitrate to N_2_, despite the fact that a greater energy yield would be achieved by catalysis of the entire pathways. Specifically, many organisms appear to be able to mediate a single step, fewer would be able to carry out two steps, and very few seem to be able to conduct three or more sequential redox transformations (Fig. 5). We do not attribute this finding to genome incompleteness because, even with conservative estimates of genome completeness^39^ (Supplementary Data 4,5), the probability that genes were consistently missed for steps in sulfur oxidation in 319 organisms and for denitrification in 330 organisms is <10^−50^ and <10^−16^, respectively (assuming a simple hypergeometric distribution). Only 10 and 12 organisms appeared to have the complete set of genes for sulfur oxidation and denitrification pathways, respectively. Based on these analyses, we conclude that use of the byproducts of the metabolism of one organism by another organism is prominent in subsurface microbial communities.

## Discussion

Microbial communities across various environments have been documented to contain thousands of different species, most of which occur at low abundance, and thus are members of the ‘rare biosphere'^20^. Because rare organisms are difficult to characterize genomically, the overall functioning of microbial communities has remained largely unknown. In this study, we demonstrate the ability to genomically describe thousands of microorganisms from a single ecosystem and bring to light aspects of the microbial community metabolic network. In addition, we defined the metabolic capacities of 1,297 organisms represented by 2,540 genomes. We show that metabolic plasticity involving the use of multiple electron donors and acceptors appears to be extremely common in microorganisms in the studied terrestrial subsurface system. A wide metabolic repertoire is likely to be important in the face of the natural environmental perturbations that occur at this site, such as seasonal snowmelt-induced fluctuations in the water table that move the oxic/anoxic interface.

In spite of redox metabolic plasticity, we found that the majority of organisms probably lack the ability to perform multiple sequential redox transformations within a pathway. This result expands on prior research that has described syntrophic interactions^4^,^40^,^41^. Thus, it appears that organisms often work in cohorts to turn biogeochemical cycles. Further, the organisms that mediate individual reaction steps display a multitude of combinations of metabolic traits, and different organisms proliferate as conditions change (Fig. 3; Supplementary Data 1 and 9). Thus, selection for different organisms to carry out specific steps in redox pathways has the potential to change the ways in which biogeochemical cycles are cross-linked. Metabolic handoffs to a wide variety of potential recipients, in combination with the potential for cycles within cycles, provide very high levels of complexity and flexibility. This modular ‘plug and play' strategy enables an enormous variety of system configurations and likely confers ecosystem resilience in the face of perturbation.

**Figure 6:**
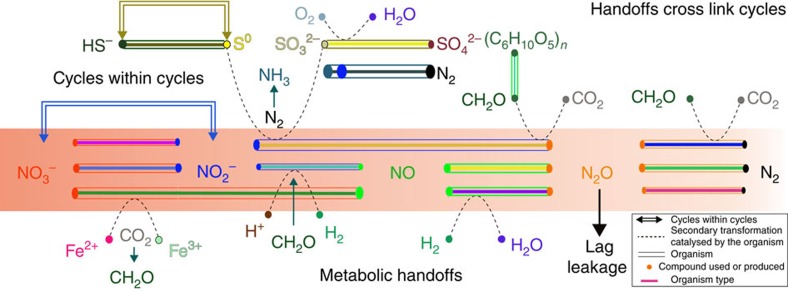
Schematic diagram illustrating the concept of metabolic handoffs and some potential consequences. Individual organisms are shown as rods. Resources inferred to be used or produced by an organism are indicated as coloured dots. Based on Supplementary Data 9, multiple organisms are potentially able to carry out specific steps, and some may be capable of more than one step. The red gradient indicates a pulse of nitrate introduced into the system. A ‘handoff' occurs when a compound produced by one organism is used by another. ‘Lag leakage' refers to the possibility that a compound moves out of the local environment (for example, N_2_O as a gas) because organisms that use it are not active at the time that its production was initiated. ‘Cycles within cycles' refers to the possibility of a sub-cycle occurring within a particular biogeochemical cycle.

Recognition of the importance of metabolic handoffs motivates new thinking about how biogeochemical processes should be modelled. Specifically, based on genomic information, individual reaction steps should be explicitly assigned to different organisms. Although this will increase model complexity and require detailed consideration of fluxes, such modifications will be essential to capture effects that can arise from metabolic handoffs, such as ‘leakage' of reaction intermediates following perturbations (Fig. 6). Leakage is likely when ecosystem discordance arises from lags in activation of microbial community members responsible for sequential steps in a biogeochemical cycle. This is analogous to the uncoupling that occurs when climate warming causes early flowering that is out of sync with insect hatching, leading to pollination failures^42^. Such phenomena are little known in microbial ecosystems, but could give rise to large fluxes of climate-relevant intermediate compounds. Examples include pulses of N_2_O following influx of ammonium-rich water^43^ or decrease in oxygen availability^44^.

Another important finding, from the perspective of development of both conceptual and quantitative models of biogeochemical processes, is the possibility of ‘cycles within cycles'. These could short-circuit the elemental cycles as they are traditionally conceived^45^ (for example, where the most reduced form, for example, S^2−^, N^3−^, is presumed to be converted to the most oxidized form, S^6+^, N^5+^ and vice versa). For example, we conclude that the inter-conversion of elemental sulfur and sulfide may be a prominent cycle within the larger sulfur cycle in this system. A similar phenomenon could also occur in the nitrogen cycle, when nitrate is reduced to nitrite by bacteria that have no further capacity for denitrification^46^, resulting in a substrate that could be oxidized back to nitrate by nitrite oxidizers.

We observed no correlation between the number or relative abundance of organisms mediating a particular step of a pathway and the total energy yields associated with that step (Supplementary Data 11). This would suggest that thermodynamic considerations alone do not control selection for the set of pathway steps that occur in organisms.

The trait distribution data (Fig. 4) highlight an example of where a cycle occurs within a larger cycle: the oxidation of sulfide to elemental sulfur, which can be converted back to sulfide rather than oxidized to sulfite and sulfate. The direct oxidation of sulfide (S^2−^) to elemental sulfur (S^0^) is mediated by two different enzymes, sulfide:quinone oxidoreductase (*sqr*)^47^ and flavocytochrome *c* sulfide dehydrogenase (*fcc*)^48^, which were present in 11% (groundwater) and 27% (sediment) of the recovered genomes. Elemental sulfur may also be produced as a byproduct of thiosulfate disproportionation by the sox enzyme system if *soxCD* are lacking^49^. Significantly, genes for elemental sulfur reduction were present in 17% (groundwater) and 22% (sediment) of the genomes, whereas the capacity for elemental sulfur oxidation was present in only 4% (groundwater) and 13% (sediment) of the genomes.

The tremendous novelty of microorganisms observed in the aquifer ecosystem highlights the potential for biological discovery in the terrestrial subsurface. Given the novel phylogenetic diversity of the studied organisms, the genomes reported here represent a vast treasure-trove that could be mined for biotechnological applications and for potential strategies for genome-enabled cultivation of novel organisms. The findings relating to metabolic network topology will guide future *in silico* studies of inter-organism metabolic networks^50^, and may have application in trait-based ecosystem models that are needed to predict the impacts of changing environmental conditions on biogeochemical cycles^51^.

## Methods

### Sampling

Groundwater and sediment samples were collected from an aquifer adjacent to the Colorado River near Rifle, CO, USA, at the Rifle Integrated Field Research site.

Sediment samples were collected from the ‘RBG' field experiment carried out in 2007. A sediment core was drilled at the location of well D04 (elevation: 1,618 m; 39° 31′ 44″ N, 107° 46′ 19″ W) and alluvial sediments with visible organic matter were collected from 4, 5 and 6 m below the surface (Fig. 1; Supplementary Data 1).

Groundwater samples were collected from three different field experiments: six sampling time points across the duration of acetate amendment (A–F); four sampling time points across the duration of oxygen injection (A–D); and two sampling time points from natural high- and low-oxygen conditions in the groundwater), driven by fluctuations in the water table at the site. Aquifer well CD-01 (elevation: 1,618 m; 39° 31′ 45″ N, 107° 46′ 20″ W) was monitored as part of a 95-day acetate amendment experiment during which acetate was added to the aquifer (target concentration of 15 μM). Following this experiment, aquifer well CD-01 was monitored as part of a 126-day oxygen injection experiment where oxygen-saturated water was injected into the aquifer (Fig. 1, Supplementary Data 1).

Aquifer well FP-101 (elevation: 1,618 m; 39° 32′ 5″ N, 107° 46′ 57″ W) was sampled during two specific time points characterized by high and low oxygen in the groundwater (Fig. 1; Supplementary Data 1). All groundwater samples were collected from 5 m below the ground surface by serial filtration onto 1.2, 0.2 and 0.1 μm filters (Supor disc filters; Pall Corporation, Port Washington, NY, USA). All sediment samples were frozen on site, while groundwater samples were preserved in RNAlater (Thermo Fisher Scientific, Waltham, MA, USA).

### Geochemical measurements

Geochemical measurements were performed on samples collected from a depth of 5 m. Water quality parameters including pH and dissolved oxygen were measured using multi-parameter sondes that were calibrated at regular intervals (YSI Inc., Yellow Springs, OH, USA). Acetate, chloride, nitrate, nitrite, thiosulfate and sulfate were measured using an ion chromatograph (ICS-1000, Dionex Corporation, Sunnyvale, CA) equipped with an AS-22 column^52^. Fe (II) and sulfide concentrations were measured using Phenanthroline and Methylene Blue colorimetric methods, respectively (Hach Company, Loveland, CO, USA). Dissolved gases in groundwater were measured using the AM20GAx method using Gas Chromatography Mass Spectrometry (Supplementary Data 1). Detailed geochemical data are publicly available from http://rifleifrc.org/geochemicaldata.

### DNA extraction and sequencing

Thirty-three samples from sediment and groundwater spanning 15 geochemical conditions were chosen for metagenomic analysis.

For the 30 groundwater samples, DNA was extracted from ∼1.5 g of each frozen filter using the PowerSoil DNA Isolation kit (MoBio Laboratories Inc., Carlsbad, CA, USA) with modifications as follows: DNA was concentrated by sodium acetate/ethanol precipitation with glycogen, followed by elution in 50 μl Tris buffer.

For the three individual sediment samples, DNA was extracted from 10 different thawed samples from the same depth to account for heterogeneity (7–14 g each) using the PowerMax Soil DNA Isolation kit (MoBio Laboratories Inc., Carlsbad, CA, USA) with the following modification to the manufacturer's instructions: samples were vortexed at maximum speed for an additional 3 min in the SDS reagent, and then incubated for 30 min at 60 °C in lieu of extended bead beating. DNA was concentrated by sodium acetate/ethanol precipitation with glycogen, followed by precipitation in 50 μl Tris buffer. Finally, all 10 replicate DNA samples were pooled together.

Metagenomic library preparation and DNA sequencing were conducted at the DOE Joint Genome Institute. DNA was sequenced on the Illumina HiSeq 2000 platform, producing 150 bp paired reads with a targeted insert size of 500 bp. Raw sequence data were processed using the Illumina CASAVA pipeline version 1.8. All reads were trimmed based on quality scores using the adaptive read trimmer, Sickle (https://github.com/najoshi/sickle; default parameters).

### Metagenomic assembly and binning

The 33 individual samples were each assembled *de novo* to obtain 33 separate assemblies. Assemblies were performed using IDBA-UD^53^ with the following parameters: --mink 40, --maxk 100, --step 20, --min_contig 500. Sequencing coverage was determined for each assembled scaffold by mapping reads from the sample to the assembly using Bowtie2 (ref. 54). All resulting scaffolds were clustered into genome bins using multiple algorithms. First, scaffolds were binned on the basis of % GC content, differential coverage abundance patterns across all 33 samples using ABAWACA^17^, and taxonomic affiliation. Scaffolds that did not associate with any cluster using this method were binned based on tetranucleotide frequency using Emergent Self-Organizing Maps (ESOM)^24^. All tetramers containing start and stop codons were removed prior to ESOM analysis as described previously^55^. The RBG13 sample (representing sediments from a depth of 3 m) could not be resolved by ABAWACA and was binned solely by tetranucleotide ESOM. All genomic bins were manually inspected within ggKbase (http://ggkbase.berkeley.edu/2500-curated-genomes/organisms). Twenty high-quality genomes chosen at random were clustered using ESOM on the basis of tetranucleotide composition for visual validation (Supplementary Fig. 3).

### Genome curation and completeness assessment

Sequence reads were mapped to all genomic scaffolds to identify assembly and scaffolding errors. Scaffolding errors typically occurred in short regions where two contigs had been erroneously scaffolded. These regions were identified and repaired as previously described^17^. In brief, errors were detected as regions with zero coverage after excluding reads mapped to the assembly with ≤2 mismatches. Reads mapped to a 1 kb region flanking the misassembly were collected and reassembled with Velvet^56^ to attempt to correct the error. Regions that could not be corrected were replaced with Ns. In cases where no paired reads spanned the detected error, the scaffolds were broken.

Genome completeness for bacteria was estimated using 43 universal single-copy genes (SCGs) that represent a subset of a previously reported list^39^ (Supplementary Data 4). The reduced set was selected due to the large proportion of CPR that either lack a number of SCG, or whose SCG are too divergent to be predicted accurately^17^. Genome completeness for archaea was estimated using 38 SCGs as described elsewhere^57^ (Supplementary Data 5).

High-quality genomes with ≤8 scaffolds were selected for genome finishing. Sixty-nine genomes passed these thresholds. Genomic scaffolds were extended by mapping reads to the end of contigs, searching for overlapping regions and for paired-read connections between contigs. Genomes were considered complete if they were circular and free of assembly errors and gaps (Ns). Read mapping for all complete genomes was performed using Bowtie2 (ref. 54) and the complete mapped read alignments were manually inspected by visualization in Geneious^58^ to ensure the absence of misassemblies by looking for discordant read pairs and zero coverage regions.

### Annotation

Open reading frames (ORFs) were predicted on genomic scaffolds using the metagenome mode of Prodigal^59^. Predicted ORFs were annotated using USEARCH (-ublast; http://drive5.com/usearch/)^37^ to search all predicted ORFs against Uniref90 (ref. 33), KEGG^32^ and an in-house database containing genomes from CPR organisms and other novel genomes from ongoing projects^7^,^13^,^14^,^15^,^16^,^17^,^22^,^60^,^61^.

### Determination of genome redundancy

Genomes were dereplicated by first generating an alignment of all scaffolds within one genome individually against scaffolds of all other bins using NUCmer^62^ at 98% nucleotide level or greater. Genomes were then grouped at >50% similarity level and the best representative was chosen based on a scoring system of SCGs: score=number of archaeal or bacterial SCGs−2 × number of multiple SCGs. In case of a tie, the genome with the greatest nucleotide information was chosen.

### Genome coverage

Read mapping for calculation of genome coverage was estimated by mapping reads against assembled scaffolds using Bowtie2 (ref. 54) with default parameters. Sample-specific genome relative abundance was calculated by normalizing for differences in read counts between samples.

### Bacterial community composition

Bacterial community composition was also determined using ribosomal protein S3 (RpS3) in order to enable comparisons of abundance of organisms for which draft genomes could not be assembled. A total of 15,247 RpS3 sequences assembled at the Rifle site to date (January 2016) were clustered at 99% with USEARCH^37^. Read mapping of all 33 individual samples was performed using Bowtie2 (ref. 54) with the following parameters (--very-sensitive --all). BlastP of the RpS3 clusters against the RpS3 genes identified on the 1,297 non-redundant genomes was performed to identify clusters with high-quality genomes from our study.

### Phylogenetic analyses using RPs and 16S rRNA

Phylogenetic analysis was performed using two different markers, the 16S rRNA gene (SSU) and a syntenic block of 16 universal RPs (L2-L6, L14-L16, L18, L22, L24, S3, S8, S10, S17 and S19). Although both methods were used for validation of phylogeny wherever possible, RPs were encountered more frequently on genomes than SSU, as observed previously^61^.

Each RP was aligned along with reference sequences using MUSCLE^63^ with default parameters. Individual RP alignments were concatenated in Geneious version 7 (ref. 58). All columns with >97% gaps were removed before further analyses. In total, the alignment of 5,969 sequences spanned 3,068 columns. Phylogenetic analysis of RP was inferred by RAxML^64^ implemented by the CIPRES Science Gateway^65^. RAxML was called as follows:

raxmlHPC-HYBRID -T 4 -s input -N autoMRE -n result -f a -p 12345 -x 12345 -m PROTCATLG. Archaea were included to the root the tree. This analysis required 4,317 computational hours, and a total of 156 bootstrapped replicates were sampled before being stopped automatically by the autoMRE algorithm. The complete RP tree is available in nexus format as Supplementary Data 12.

For SSU analysis, 573 16S rRNA genes representing non-redundant genomes were aligned with 4,673 bacterial, archaeal and eukaryotic reference sequences with the SINA aligner^66^ using the SILVA web interface^67^ with default parameters. 16S rRNA genes could not be linked to all 1,297 genomes since rRNA regions in scaffolds often fragment and are hence difficult to bin^68^. All introns in 16S rRNA genes were removed as described previously^17^. All columns with >95% gaps were removed and the final alignment spanned 1,626 nucleotides. Phylogenetic analysis of the 16S rRNA gene SSU was inferred by RAxML^64^. RAxML was called as follows:

raxmlHPC-PTHREADS -f a -s input -n result -m GTRGAMMA -x 12345 -# autoMRE -p 12345 -T 4. Eukarya were included as the root for the tree. A total of 300 bootstrapped replicates were sampled before being stopped automatically by the autoMRE algorithm. The complete 16S rRNA tree is available in nexus format as Supplementary Data 13.

Phylogenetic trees were visualized with figtree v1.2.2 (http://tree.bio.ed.ac.uk/software/figtree/).

### Identification of novel phylum-level lineages

Novel phylum-level lineages were proposed on the basis of three conditions. First, 16S rRNA genes had a pairwise identity less than ∼75% with known phylum-level lineages and formed a monophyletic clade. This threshold for difference in 16S rRNA gene identity between phylum-level lineages has been proposed previously^27^. Second, RP phylogeny indicates these genomes form a monophyletic clade. And third, high-quality draft or near-complete genomes were available for these phylum-level lineages. We propose names for these newly described phylum-level lineages based on eminent microbiologists and current University of California, Berkeley microbiology faculty (Table 1).

### Metabolic potential analysis

Genome-specific metabolic potential was determined by (1) searching all predicted ORFs in a genome with Pfam^35^, TIGRfam^34^, Panther^69^ and custom HMM profiles (Supplementary Data 8 and 12) of marker genes for specific pathways using hmmscan^36^, and (2) assessment of complete pathways for metabolic transformations using ggKbase. For generation of custom HMM profiles, references for each marker gene were aligned using MUSCLE with default parameters followed by manually trimming the start and ends of the alignment. The alignment was converted into Stockholm format and databases were built using hmmscan^36^. For Rubisco and hydrogenases^70^, different hmm databases were constructed for each distinct group. For HMM searches against TIGRfam, all hits above the preset noise cutoff were considered for manual inspection. Individual cutoffs for all HMMs were determined by manual inspection and are listed in Supplementary Data 14.

In ggKbase, lists for specific metabolic pathways were generated by searching for specific keywords in gene annotations. Coupling the genome abundance to metabolic traits allowed the simultaneous assessment of all 2,540 genomes assembled in this study. All custom HMM profiles used in this study are publicly available from https://github.com/banfieldlab.

### Data availability

DNA sequences (genomes and raw sequence reads) have been deposited in NCBI BioProject database with accession code PRJNA288027. NCBI Genbank accession numbers for individual genomes are listed in Supplementary Data 3. Genomes are also available through ggKbase: http://ggkbase.berkeley.edu/2500-curated-genomes/organisms (ggKbase is a ‘live' site, genomes may be updated after publication). Detailed geochemical data are publicly available from http://rifleifrc.org/geochemicaldata. Hmm databases used in this study are available from https://github.com/banfieldlab/metabolic-hmms. The authors declare that all other data supporting the findings of this study are available within the article and its supplementary information files, or from the corresponding author on request.

## Additional information

**How to cite this article:** Anantharaman, K. *et al*. Thousands of microbial genomes shed light on interconnected biogeochemical processes in an aquifer system. *Nat. Commun.* **7,** 13219 doi: 10.1038/ncomms13219 (2016).

## Supplementary Material

Supplementary Information Supplementary Figures 1-3.

Supplementary Movie 1 Animation (gif) showing variation in abundance at the genomelevel of organisms across the fifteen geochemical conditions sampled. All genome coverage was normalized to the low oxygen groundwater sample. For groundwater, only samples collected on the 0.2 μm filter were considered. Colors represent different experiments/condtions as follows: Red: Low O2 groundwater; Black: High O2 groundwater; Blue: Acetate stimulation of groundwater; Green: Oxygen stimulation of groundwater; Purple: Natural unamended sediments.

Supplementary Movie 2 Animation (gif) showing variation in abundance at the phylumlevel of organisms across the fifteen geochemical conditions sampled. All genome coverage was normalized to the low oxygen groundwater sample. For groundwater, only samples collected on the 0.2 μm filter were considered. Colors represent different experiments/condtions as follows: Red: Low O2 groundwater; Black: High O2 groundwater; Blue: Acetate stimulation of groundwater; Green: Oxygen stimulation of groundwater; Purple: Natural unamended sediments.

Supplementary Movie 3 Animation (gif) showing variation in abundance at the genomelevel of organisms with the capacity to utilize oxygen across the fifteen geochemical conditions sampled. All genome coverage was normalized to the low oxygen groundwater sample. For groundwater, only samples collected on the 0.2 μm filter were considered. Colors represent different experiments/condtions as follows: Red: Low O2 groundwater; Black: High O2 groundwater; Blue: Acetate stimulation of groundwater; Green: Oxygen stimulation of groundwater; Purple: Natural unamended sediments.

Supplementary Movie 4 Animation (gif) showing variation in abundance at the genomelevel of organisms during the oxygen stimulation experiment. Only samples collected on the 0.2 μm filter were considered.

Supplementary Movie 5 Animation (gif) showing variation in abundance at the genomelevel of organisms during the acetate stimulation experiment. Only samples collected on the 0.2 μm filter were considered.

Supplementary Data 1 Geochemical measurements from the four different field experiments conducted at the Rifle IFRC site. Data is also available for download from http://www.rifleifrc.org/geochemicaldata.

Supplementary Data 2 Metagenomics sequencing and assembly statistics.

Supplementary Data 3 Details of genomes reconstructed from sediments and groundwater. Coverage of the genomes was calculated from read-mapping to the source sample.

Supplementary Data 4 Single Copy Gene (SCG) analysis and genome completion estimates for sediment and groundwater bacterial genomes.

Supplementary Data 5 Single Copy Gene (SCG) analysis and genome completion estimates for sediment and groundwater archaeal genomes.

Supplementary Data 6 Non-redundant genomes and genome clusters. Cluster winners represent non-redundant genomes.

Supplementary Data 7 Genomes from different bacterial and archaeal phyla from sediment and groundwater. All complete genomes have been manually curated and are circular. Genome completeness was estimated from the percentage of 43 single copy genes (SCGs) identified in each genome.

Supplementary Data 8 Abundance of the genomes measured as relative coverage (x) in the microbial community across all thirty-three samples collected from Rifle groundwater and sediment. Coverage of each genome was determined by read mapping of scaffolds to each individual sample. Cells are colored in accordance with abundance in the microbial community as follows: Red: 25th percentile, Orange: 75th percentile, Green: 95th percentile.

Supplementary Data 9 Phylogeny and Trait Analysis of sediment and groundwater genomes. Individual traits were determined by hmm searches of marker genes and ggKbase lists for specific pathways associated with the traits. Presence/Absence of traits are shown in binary form - Presence: 1 (Green), Absence: 0 (Red).

Supplementary Data 10 Elemental cycling associated with sediment and groundwater associated genomes as described in Figure 1. Presence/Absence of Elemental cycling mechanisms are shown in binary form - Presence: 1 (Green), Absence: 0 (Red).

Supplementary Data 11 Standard Gibbs free energy of reactions involved in microbial sulfur and nitrogen cycling. Only groundwater samples collected on the 0.2 μm filter were considered. All denitrification reactions are shown with hydrogen as the electron donor for consistency. Total number and Abundance of organisms assicated with denitrification involves all potential electron donors.

Supplementary Data 12 RP phylogeny in Nexus format.

Supplementary Data 13 16S rRNA gene phylogeny in Nexus format.

Supplementary Data 14 Hmm databases and respective cutoff scores used for validation of metabolic marker genes. Rows in red were excluded from analysis.

## Figures and Tables

**Table 1:** Proposed names for newly described phyla.

Code	Proposed phylum name	Named after	Institution/explanation
RIF1	*Candidatus* Firestonebacteria	Mary K. Firestone	University of California, Berkeley
RIF2	*Candidatus* Lindowbacteria	Steven E. Lindow	University of California, Berkeley
RIF3	*Candidatus* Schekmanbacteria	Randy W. Schekman	University of California, Berkeley
RIF4	*Candidatus* Kerfeldbacteria	Cheryl A. Kerfeld	University of California, Berkeley
RIF5	*Candidatus* Glassbacteria	N. Louise Glass	University of California, Berkeley
RIF6	*Candidatus* Komeilibacteria	Arash Komeili	University of California, Berkeley
RIF7	*Candidatus* Raymondbacteria	Kenneth N. Raymond	University of California, Berkeley
RIF8	*Candidatus* Coatesbacteria	John D. Coates	University of California, Berkeley
RIF9	*Candidatus* Andersenbacteria	Gary L. Andersen	Lawrence Berkeley National Laboratory
RIF10	*Candidatus* Ryanbacteria	Kathleen R. Ryan	University of California, Berkeley
RIF11	*Candidatus* Niyogibacteria	Krishna K. Niyogi	University of California, Berkeley
RIF12	*Candidatus* Tagabacteria	Michiko E. Taga	University of California, Berkeley
RIF13	*Candidatus* Terrybacteria	Norman Terry	University of California, Berkeley
RIF14	*Candidatus* Vogelbacteria	John P. Vogel	University of California, Berkeley
RIF15	*Candidatus* Zambryskibacteria	Patricia C. Zambryski	University of California, Berkeley
RIF16	*Candidatus* Taylorbacteria	John W. Taylor	University of California, Berkeley
RIF17	*Candidatus* Sungbacteria	Z. Renee Sung	University of California, Berkeley
RIF18	*Candidatus* Brennerbacteria	Steven E. Brenner	University of California, Berkeley
RIF19	*Candidatus* Spechtbacteria	Chelsea D. Specht	University of California, Berkeley
RIF20	*Candidatus* Staskawiczbacteria	Brian J. Staskawicz	University of California, Berkeley
RIF21	*Candidatus* Wildermuthbacteria	Mary C. Wildermuth	University of California, Berkeley
RIF22	*Candidatus* Portnoybacteria	Daniel A. Portnoy	University of California, Berkeley
RIF23	*Candidatus* Muproteobacteria	Greek letter ‘Mu' (μ)	In continuation of the practice of naming lineages within *Proteobacteria* with greek letters, we suggest 'Mu'.
RIF24	*Candidatus* Lambdaproteobacteria	Greek letter 'Lambda' (λ)	In continuation of the practice of naming lineages within *Proteobacteria* with greek letters, we suggest 'Lambda'.
RIF25	*Candidatus* Fischerbacteria	Robert L. Fischer	University of California, Berkeley
RIF26	*Candidatus* Delongbacteria	Edward F. DeLong	University of Hawaii, Manoa
RIF27	*Candidatus* Handelsmanbacteria	Jo E. Handelsman	Yale University
RIF28	*Candidatus* Eisenbacteria	Jonathan A. Eisen	University of California, Davis
RIF29	*Candidatus* Edwardsbacteria	Katrina J. Edwards	University of Southern California
RIF30	*Candidatus* Margulisbacteria	Lynn Margulis	University of Massachusetts at Amherst
RIF31	*Candidatus* Fraserbacteria	Claire M. Fraser	University of Maryland
RIF32	*Candidatus* Riflebacteria	Rifle	Sampling site for this study
RIF33	*Candidatus* Wallbacteria	Judy D. Wall	University of Missouri
RIF34	*Candidatus* Woykebacteria	Tanja Woyke	DOE Joint Genome Institute
RIF35	*Candidatus* Blackburnbacteria	Elizabeth H. Blackburn	University of California, San Francisco
RIF36	*Candidatus* Chisholmbacteria	Sallie W. Chisholm	Massachusetts Institute of Technology
RIF37	*Candidatus* Buchananbacteria	Bob B. Buchanan	University of California, Berkeley
RIF38	*Candidatus* Jacksonbacteria	Andrew O. Jackson	University of California, Berkeley
RIF39	*Candidatus* Veblenbacteria	David R. Veblen	Johns Hopkins University
RIF40	*Candidatus* Nealsonbacteria	Kenneth H. Nealson	University of Southern California
RIF41	*Candidatus* Colwellbacteria	Rita R. Colwell	University of Maryland
RIF42	*Candidatus* Liptonbacteria	Mary S. Lipton	Pacific Northwest National Laboratory
RIF43	*Candidatus* Harrisonbacteria	Susan T.L. Harrison	University of Cape Town
RIF44	*Candidatus* Yonathbacteria	Ada E. Yonath	Weizmann Institute of Science
RIF45	*Candidatus* Lloydbacteria	Jonathan R. Lloyd	University of Manchester
RIF46	*Candidatus* Abawacabacteria	Abawaca	Program used for metagenomic binning
SM2F11	*Candidatus* Doudnabacteria	Jennifer A. Doudna	University of California, Berkeley
