# Supplementary material for: Thousands of microbial genomes shed light on interconnected biogeochemical processes in an aquifer system
Source: Nat Commun. 2016 Oct 24;7:13219. doi: 10.1038/ncomms13219 (PMC5079060; doi:10.1038/ncomms13219)
Supplement: Supplementary Information — Supplementary Figures 1-3. [file ncomms13219-s1.pdf]

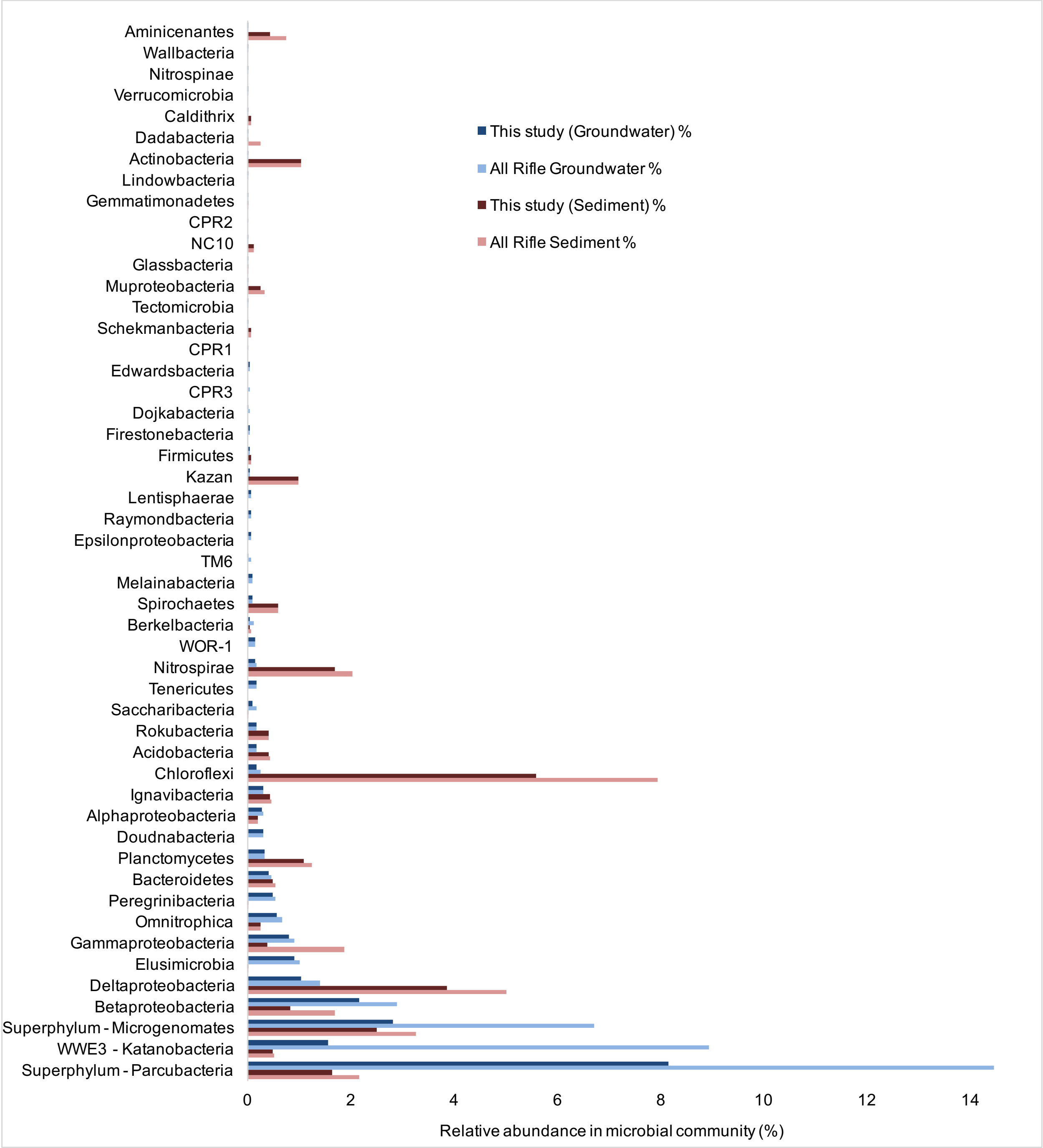

Supplementary Figure 1: Comparison of relative abundances of phyla described in this study with all high-quality genomes recovered from groundwater and sediment. Abundances were calculated using the ribosomal protein S3 (RpS3) clustered at 99% and linked to individual genomes. Only top 30 phyla are shown.

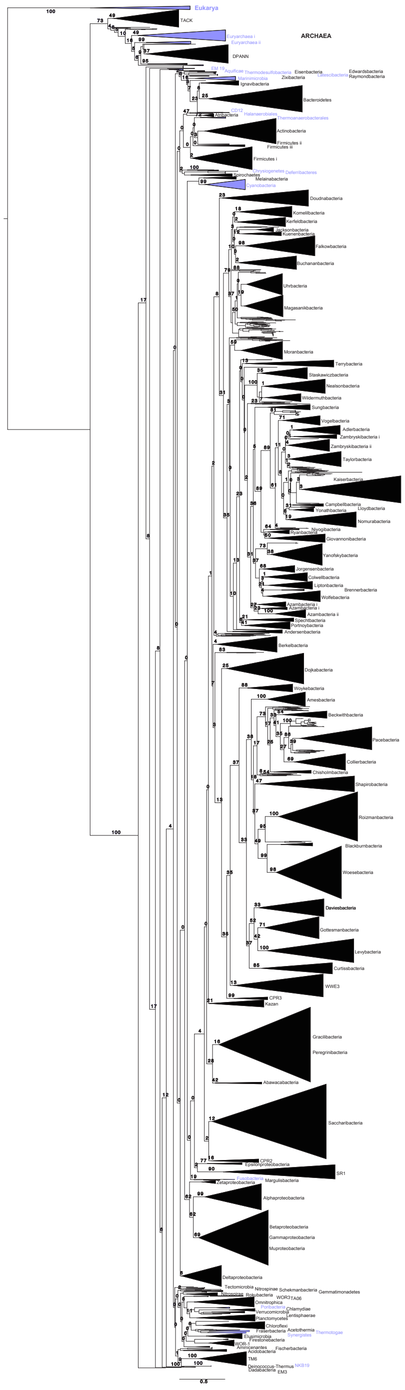

Supplementary Figure 2. Phylogenetic analysis of 16S rRNA genes inferred by maximum-likelihood implemented with RAxML. The tree is collapsed at the phylum-level. Colors of the wedges indicate the following: Black: Phyla identified at Rifle; Blue: Phyla not identified at Rifle. Not all are bootstrap values are shown. The phylogenetic inference configurations with detailed branch support values are provided in nexus format in Supplementary Data 13

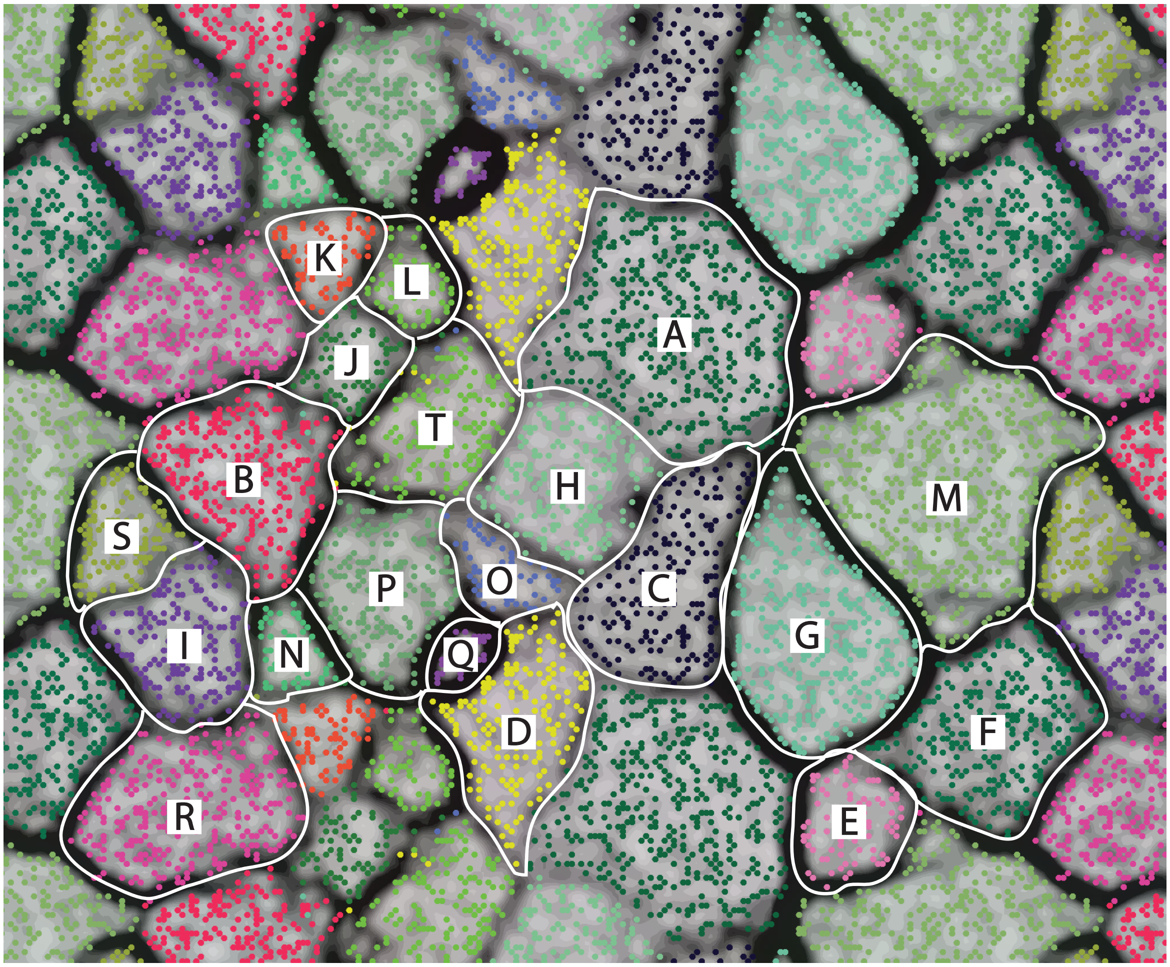

Supplementary Figure 3. Validation of 20 high-quality draft genomes chosen at random using tetranucleotide sequence composition. Each colored point represents a 5-10 kb genome fragment. Background color is dictated by tetranucleotide frequency variance between data points with grey indicating similarity and black demarcating different genomes. White boxes delineate the genomes. The letters indicate individual genomes as follows:
A, Bacteroidetes bacterium GWA2_40_15; B, Gallionellales bacterium GWA2_54_124 ; C, *Candidatus* Firestonebacteria bacterium GWA2_43_8; D, *Candidatus* Schekmanbacteria bacterium GWA2_38_11; E, *Omnitrophica* WOR_2 bacterium GWA2_53_43; F, Elusimicrobia bacterium GWB2_63_22; G, *Candidatus* Wallbacteria bacterium GWC2_49_35; H, *Candidatus* Melainabacteria bacterium GWF2_37_15; I, *Candidatus* Fraserbacteria bacterium RBG_16_55_9; J, *Candidatus* Doudnabacteria bacterium RIFCSPHIGHO2_01_FULL_46_24; K, *Candidatus* Doudnabacteria bacterium RIFCSPHIGHO2_01_FULL_50_11; L, candidate division Kazan bacterium RIFCSPLOWO2_01_FULL_45_19; M, Acidobacteria bacterium RIFCSPLOWO2_02_FULL_65_29; N, Candidatus Kaiserbacteria bacterium RIFCSPLOWO2_12_FULL_50_28; O, candidate division WWE3 bacterium RIFCSPLOWO2_12_FULL_36_10; P, *Sulfurimonas* sp. RIFOXYB12_FULL_35_9; Q, candidate division WS6 bacterium RIFOXYB1_FULL_33_14; R, Spirochaetes bacterium RIFOXYC1_FULL_54_7; S, *Candidatus* Peribacter riflensis; T, *Candidatus* Falkowbacteria bacterium RIFOXYD2_FULL_34_120.
