# Supplementary figures and images for: Thousands of microbial genomes shed light on interconnected biogeochemical processes in an aquifer system
Source: Nat Commun. 2016 Oct 24;7:13219. doi: 10.1038/ncomms13219 (PMC5079060; doi:10.1038/ncomms13219)

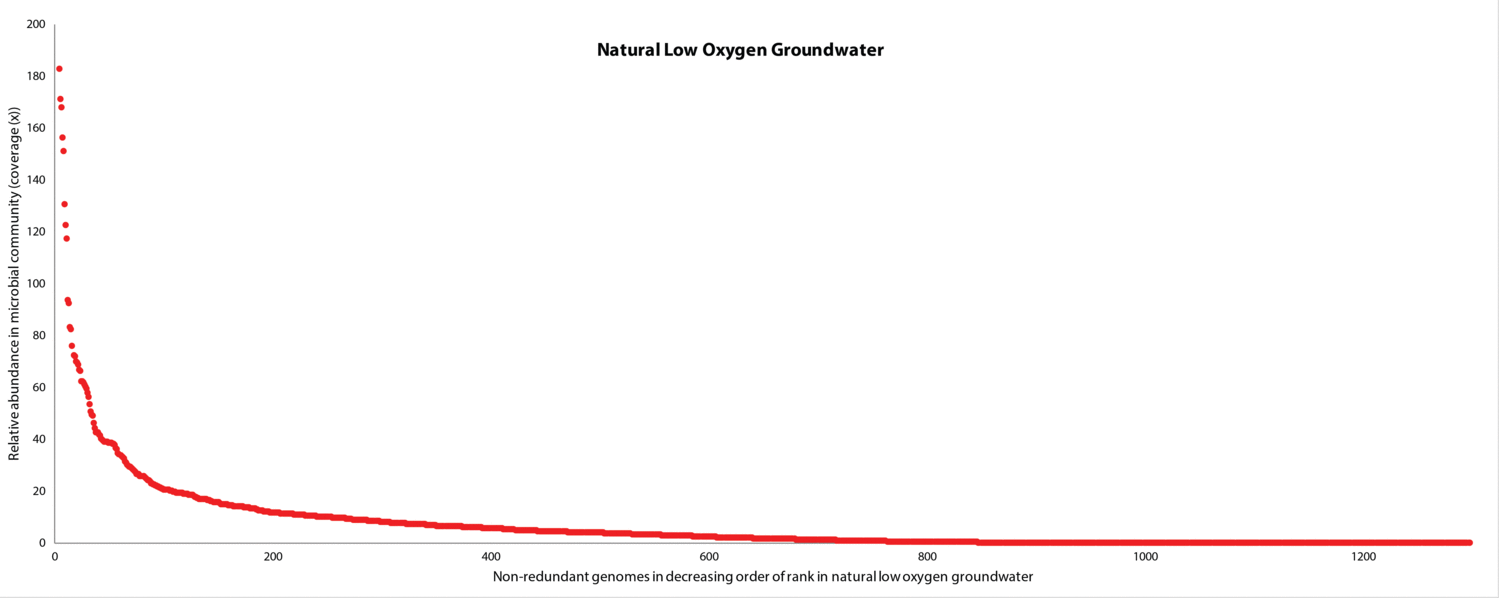

Supplement: Supplementary Movie 1 — Animation (gif) showing variation in abundance at the genomelevel of organisms across the fifteen geochemical conditions sampled. All genome coverage was normalized to the low oxygen groundwater sample. For groundwater, only samples collected on the 0.2 μm filter were considered. Colors represent different experiments/condtions as follows: Red: Low O2 groundwater; Black: High O2 groundwater; Blue: Acetate stimulation of groundwater; Green: Oxygen stimulation of groundwater; Purple: Natural unamended sediments. [file ncomms13219-s2.gif]

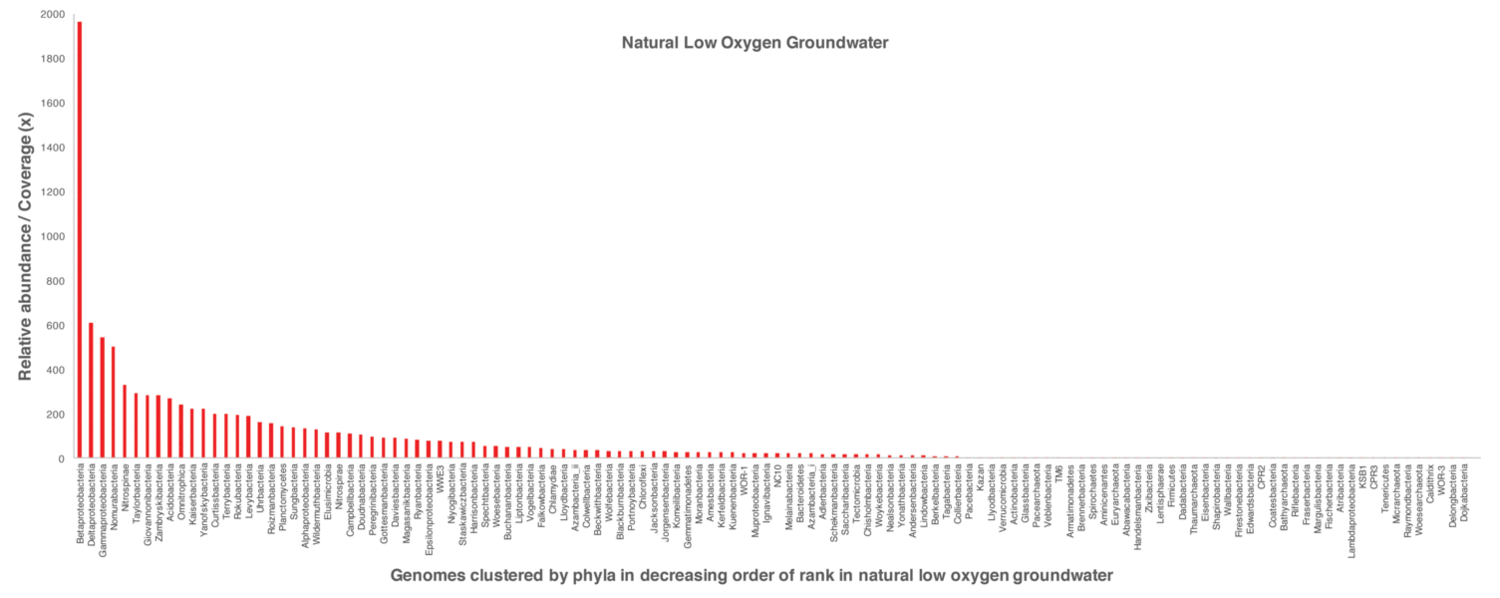

Supplement: Supplementary Movie 2 — Animation (gif) showing variation in abundance at the phylumlevel of organisms across the fifteen geochemical conditions sampled. All genome coverage was normalized to the low oxygen groundwater sample. For groundwater, only samples collected on the 0.2 μm filter were considered. Colors represent different experiments/condtions as follows: Red: Low O2 groundwater; Black: High O2 groundwater; Blue: Acetate stimulation of groundwater; Green: Oxygen stimulation of groundwater; Purple: Natural unamended sediments. [file ncomms13219-s3.gif]

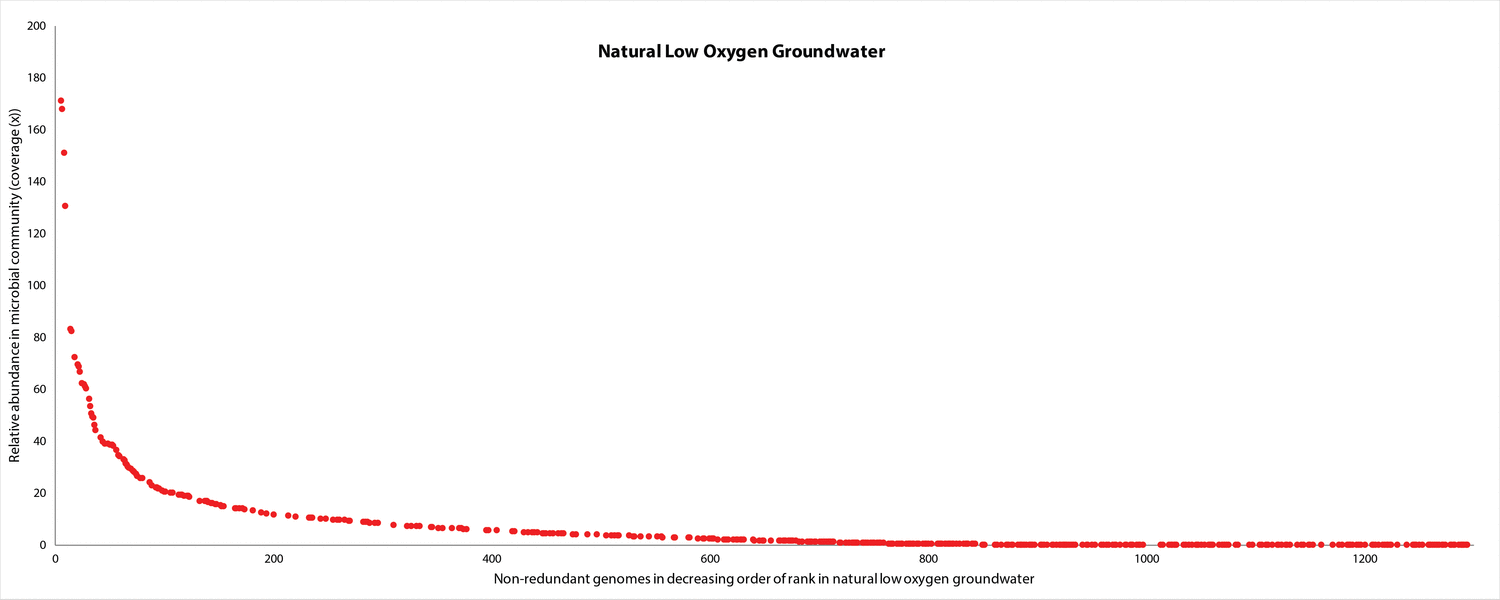

Supplement: Supplementary Movie 3 — Animation (gif) showing variation in abundance at the genomelevel of organisms with the capacity to utilize oxygen across the fifteen geochemical conditions sampled. All genome coverage was normalized to the low oxygen groundwater sample. For groundwater, only samples collected on the 0.2 μm filter were considered. Colors represent different experiments/condtions as follows: Red: Low O2 groundwater; Black: High O2 groundwater; Blue: Acetate stimulation of groundwater; Green: Oxygen stimulation of groundwater; Purple: Natural unamended sediments. [file ncomms13219-s4.gif]

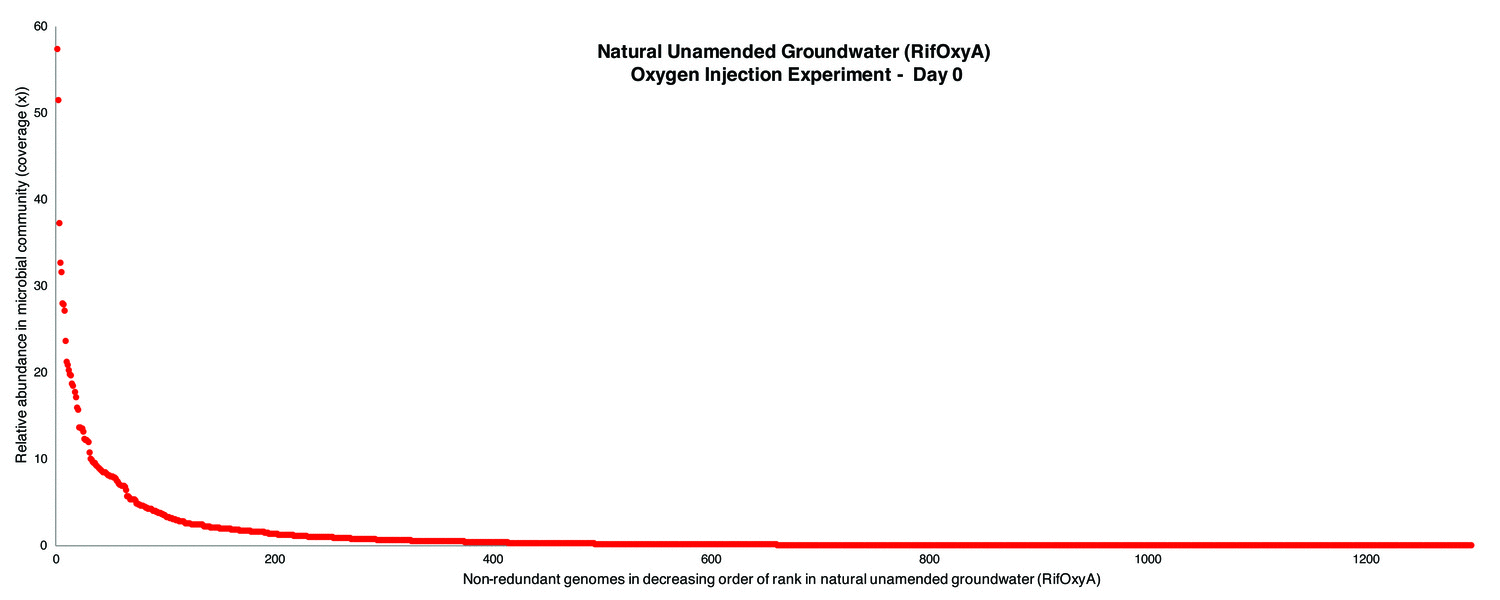

Supplement: Supplementary Movie 4 — Animation (gif) showing variation in abundance at the genomelevel of organisms during the oxygen stimulation experiment. Only samples collected on the 0.2 μm filter were considered. [file ncomms13219-s5.gif]

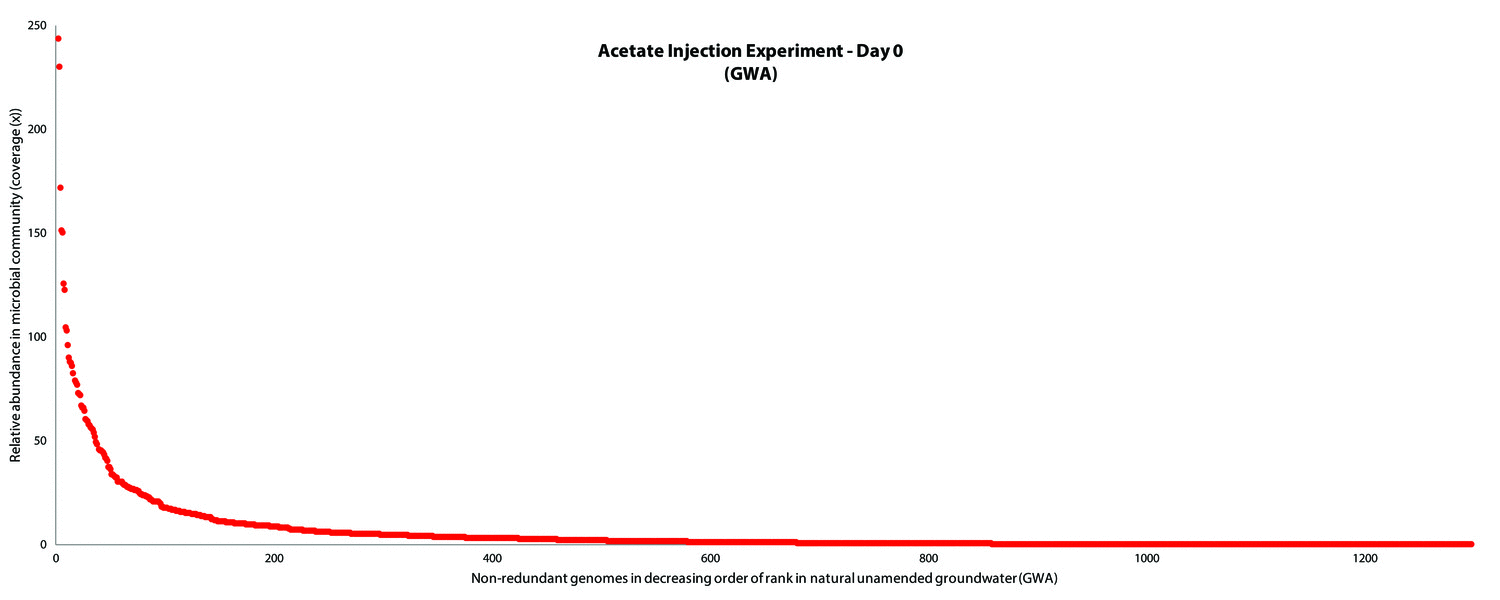

Supplement: Supplementary Movie 5 — Animation (gif) showing variation in abundance at the genomelevel of organisms during the acetate stimulation experiment. Only samples collected on the 0.2 μm filter were considered. [file ncomms13219-s6.gif]
